# Map building using helmet-mounted LiDAR for micro-mobility

**DOI:** 10.1007/s10015-022-00848-6

**Published:** 2023-01-10

**Authors:** Ibuki Yoshida, Akihiko Yoshida, Masafumi Hashimoto, Kazuhiko Takahashi

**Affiliations:** 1 Graduate School of Science and Engineering, Doshisha University, Kyotanabe, Japan; 2 Faculty of Science and Engineering, Doshisha University, Kyotanabe, Japan

**Keywords:** Helmet-mounted LiDAR, NDT SLAM, Micro-mobility, Distortion correction

## Abstract

This paper presents a point-cloud mapping method using a light detection and ranging (LiDAR) mounted on a helmet worn by a rider of micro-mobility. The distortion in LiDAR measurements, which is caused by motion and shaking of micro-mobility and rider, is corrected by estimating the pose (3D positions and attitude angles) of the helmet based on the information from normal distributions transform-based simultaneous localization and mapping (NDT SLAM) and an inertial measurement unit. A Kalman filter-based algorithm for the distortion correction is presented under the assumption that the helmet moves at nearly constant translational and angular velocities in any directions. The distortion-corrected LiDAR measurements are mapped onto an elevation map, and the measurements relating to stationary objects in the environments are extracted using the occupancy grid method. The stationary object measurements are utilized to build a point-cloud map. The experimental results in a campus road environment demonstrate the effectiveness of the proposed method.

## Introduction

Recently, many studies on active safety and self-driving of vehicles have been conducted in the field of intelligent transportation systems (ITS) [1]. In addition, last-mile delivery automation utilizing delivery robots is actively being studied in the field of transport and logistics [2]. An important technology for self-driving and active safety of vehicles and mobile robots is the building of environmental maps [3, 4]. To that end, many methods of map building have been proposed using mobile mapping systems and simultaneous localization and mapping (SLAM) [5–7]. In this paper, we focus on SLAM based on light detection and ranging (LiDAR).

We proposed a method to build three-dimensional (3D) point-cloud maps in residential environments using a car-mounted LiDAR based on the normal distributions transform (NDT)–Graph SLAM [8]. Since it is more convenient to use two-wheeled vehicles than cars to build environmental maps in narrow road environments, we also proposed a method to build 3D point-cloud maps using a LiDAR mounted on a two-wheeled vehicle [9].

To reduce carbon emissions and traffic congestion in urban cities, the use of micro-mobilities, such as bicycles, mopeds, e-scooters, and personal mobility devices, has been attracting attention for short-distance transportation [10]. Additionally, due to the impact of the novel coronavirus disease (COVID-19), there has been increased resistance to the use of transportation methods, such as crowded trains and busses. This has led to the increased use of micro-mobilities, by which the risk of infection can make lower. As the pandemic makes the transition to becoming endemic in the near future, the use of micro-mobilities will continue to increase.

Reducing the number of traffic accidents related to micro-mobilities is vital for the widespread use of micro-mobilities. As such, we are examining the map building and moving-object recognition for active safety of micro-mobilities in sidewalk and roadway environments. With micro-mobilities, the size of such devices, as well as concerns over theft, makes it difficult to permanently install multiple sensor systems as can be done in automobiles. Thus, it is more desirable to install a small removable sensor onto the handle bar of the micro-mobility or onto the helmet.

Therefore, this paper presents a method of building a 3D point-cloud environmental map using a LiDAR mounted on the rider helmet of a micro-mobility. This paper is an expansion of our previous works [8, 9]: the environmental map building methods using a LiDAR mounted on automobiles and two-wheeled vehicles. By building maps of sidewalk and roadway environments, its application to the active safety of micro-mobilities, the self-driving of delivery robots, and so on becomes possible.

There were several studies on environmental map building and self-localization using indoor SLAM, in which pedestrians wore helmets equipped with one- or two-dimensional LiDAR [11–13]. However, to the best of our knowledge, there have been no studies on map building of sidewalk and roadway environments, in which a rider of a micro-mobility wore a helmet equipped with a 3D LiDAR. Since a LiDAR mounted on the rider helmet of a micromobility suffers stronger motion and shaking than a LiDAR mounted on a pedestrian helmet, distortion appears in mapping results.

Map building algorithms usually assume that all LiDAR measurements within a scan (one rotation of laser beams on the horizontal plane) are obtained at the same time, and all measurements are thus mapped onto the world coordinate system using the same position of a LiDAR. However, the LiDAR obtains range measurements by scanning laser beams; thus, when the LiDAR moves and shakes strongly, all LiDAR measurements within a scan cannot be obtained at the same position of the LiDAR. Therefore, a large distortion appears in mapping results. To correct this distortion, the helmet’s pose has to be estimated every LiDAR measurement is obtained within a scan.

Many methods for distortion correction have been proposed in the fields of mobile robotics and ITS, in most of which linear interpolation between the poses of adjacent scans and its variant approaches was used [14–18]. However, distortion correction of LiDAR measurements in strong motion and shaking conditions still remains a significant challenge. In this paper, the Kalman filter-based distortion correction [19, 20] is utilized: the helmet’s pose is estimated using Kalman filter every LiDAR measurement is obtained within a scan. Since the Kalman filter-based localization is well used in the fields of mobile robotics and ITS, the distortion correction in a Kalman filter framework can be easily embedded into the self-localization system of the micro-mobility.

In the field of ITS, studies on helmets equipped with sensors (smart helmets) are being studied [21–23]; however, such studies have been limited to detecting alcohol for motorcycle riders, collision detection, and confirming the safety of riders following an accident.

The rest of this paper is organized as follows: In Sect. 2, the experimental system is described. In Sect. 3, NDT SLAM is summarized. In Sects. 4 and 5, methods to correct distortion in LiDAR measurements and to extract LiDAR measurements relating to stationary objects are discussed, respectively. In Sect. 6, the characteristics of the proposed method are shown through experiments, and then, the main conclusions of this paper are discussed in Sect. 7.

## Experimental system

**Fig. 1:**
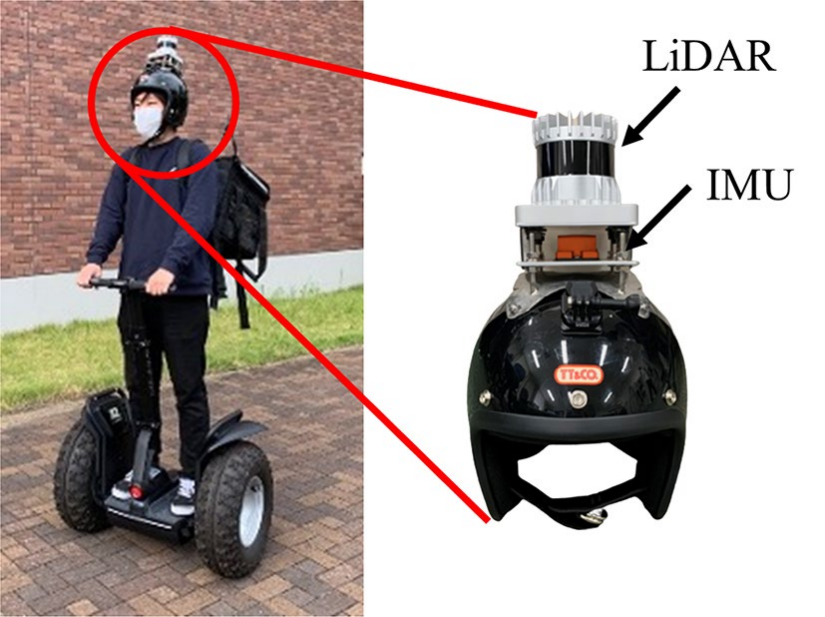
Overview of experimental smart helmet

Figure 1 shows the appearance of the smart helmet. A mechanical 64-layer LiDAR (Ouster OS0-64) and an inertial measurement unit (IMU, Xsens MTi-300) are installed on the top of the helmet. The maximum observable range for the

LiDAR is 55 m. The horizontal viewing angle is 360° with a resolution of 0.35°, and the vertical viewing angle is 90° (elevation of 45° and depression of 45°) with a resolution of 1.4°. The LiDAR obtains 1024 measurements (distance, direction and reflection intensity) every 1.56 ms (every 5.6° in the horizontal direction). Therefore, approximately 66,000 LiDAR measurements are obtained for a period (100 ms) of one rotation (360°). In this paper, one horizontal rotation (360°) of the LiDAR laser beam is called one scan.

The IMU obtains the attitude angle (roll and pitch angles) and angular velocity (roll, pitch, and yaw angular velocities) every 10 ms. The measurement error for the attitude angle is less than ± 0.3°, while that for the angular velocity is less than ± 0.2°/s. The IMU also outputs the yaw angle information. However, we do not use it because the accuracy of the yaw angle is significantly affected by magnetic disturbances in environments.

Since the weight of the LiDAR is about 0.5 kg, it is heavier and larger than a usual helmet. Therefore, there are issues in terms of practicality and usability. However, modern LiDAR technologies have been developing solid-state LiDARs [24], which are smaller, lighter, and more energy-friendly than the mechanical LiDARs used in our experiment system. Use of solid-state LiDARs would greatly improve the smart helmet in terms of practicality and usability.

## Overview of NDT SLAM

**Fig. 2:**
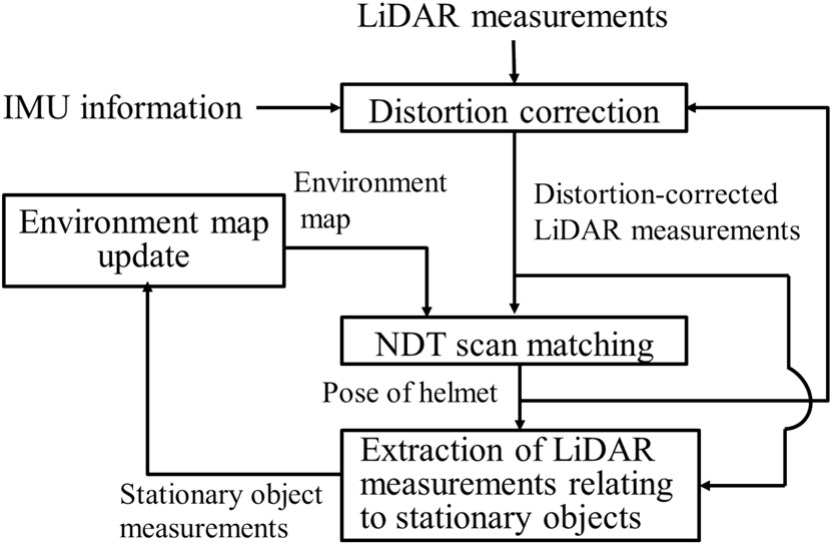
Overview of map building using NDT SLAM

Figure 2 shows the flow of environmental map building using NDT SLAM. Distortion correction and extraction of LiDAR measurements relating to stationary objects will be discussed later. In this section, NDT SLAM using distortion-corrected LiDAR measurements is summarized.

**Fig. 3:**
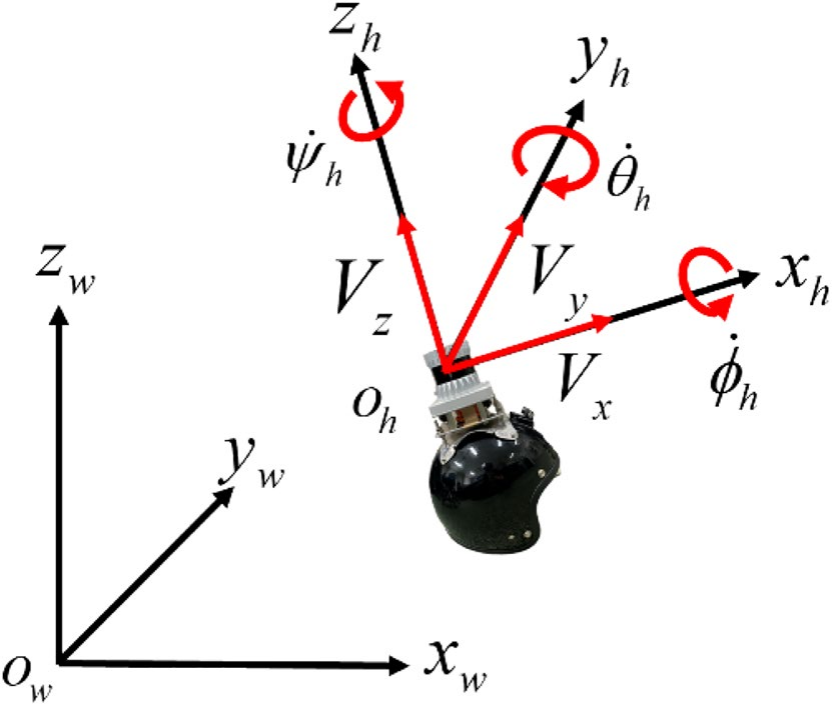
Notation related to the helmet motion

As shown in Fig. 3, let us consider a helmet coordinate system $\Sigma_{h}$ with the emitting point of the LiDAR laser as the origin *O*_*h*_, the front of the helmet as the *x*_*h*_ axis, and the zenith direction as the *z*_*h*_-axis. A 3D grid map (voxel map), each of which cell is a cube with a grid size of 0.2 m, is considered in $\Sigma_{h}$. When LiDAR measurements are obtained, they are mapped on the voxel map and downsized with a voxel grid filter. In the subsequent processing, the downsized measurements are used to calculate the self-pose of the helmet using NDT SLAM, and the LiDAR measurements prior to downsizing are used for the mapping (building of point-cloud map) in the world coordinate system $\Sigma_{w}$, which is defined as follows: First, a coordinate system $\Sigma_{1}$ is given by rotating $\Sigma_{h}$ around the *x*_*h*_ axis by $- \,\phi (0)$. Next, $\Sigma_{w}$ is determined by rotating $\Sigma_{1}$ around the *y*_1_ axis in $\Sigma_{1}$ by $- \,\theta (0)$. Here, $\phi (0)$ and $\theta (0)$ are the roll and pitch angle information, respectively, which is obtained from the IMU when the micro-mobility is at the starting point.

The 3D position of the *i*-th measurement (*i* = 1, 2,..., *n*) in $\Sigma_{h}$ is defined as ${\mathbf{p}}_{hi} = (x_{hi} ,y_{hi} ,z_{hi} )^{T}$ and that in $\Sigma_{w}$ as ${\mathbf{p}}_{i} = (x_{i} ,y_{i} ,z_{i} )^{T}$. Then, the homogeneous transformation leads to the following equation:

1 $$ \left( {\begin{array}{*{20}c} {{\mathbf{p}}_{i} } \\ 1 \\ \end{array} } \right) = {\mathbf{\rm T}}({\mathbf{X}})\left( {\begin{array}{*{20}c} {{\mathbf{p}}_{hi} } \\ 1 \\ \end{array} } \right) $$

where $(x,y,z)^{T}$ and $(\phi ,\theta ,\psi )^{T}$ in ${\mathbf{X}} = (x,y,z,\phi ,\theta ,\psi )^{T}$ are the 3D position and attitude angle (roll, pitch, and yaw angles), respectively, of the helmet in $\Sigma_{w}$. ***T***(***X***) is the following homogeneous transformation matrix:

$$ {\rm T}({\mathbf{X}}) = \left( {\begin{array}{*{20}c} {\cos \theta \cos \psi } & {\sin \phi \sin \theta \cos \psi - \cos \phi \sin \psi } & {\cos \phi \sin \theta \cos \psi + \sin \phi \sin \psi } & x \\ {\cos \theta \sin \psi } & {\sin \phi \sin \theta \sin \psi + \cos \phi \cos \psi } & {\cos \phi \sin \theta \sin \psi - \sin \phi \cos \psi } & y \\ { - \sin \theta } & {\sin \phi \cos \theta } & {\cos \phi \cos \theta } & z \\ 0 & 0 & 0 & 1 \\ \end{array} } \right) $$

In NDT SLAM, a voxel map for a cube with a cell size of 0.6 m is set in $\Sigma_{w}$. In each cell of the voxel map, a normal distributions transform is performed on the LiDAR measurements obtained up to the prior time (referred to as environmental map), and the mean and covariance of the LiDAR measurements are calculated.

By superimposing the LiDAR measurements obtained at the current time (referred to as new measurements) over the environmental map, the self-pose ***X*** of the helmet at the current time can be calculated [25]. Using the self-pose and Eq. (1), the new measurements obtained in $\Sigma_{h}$ are transformed to those in $\Sigma_{w}$ and merged with the environmental map. By repeating this process every LiDAR scan period, an environment map (3D point-cloud map) can be sequentially built.

## Distortion correction of LiDAR measurements

### Motion and observation models

Since LiDAR obtains measurements by scanning laser beams, all measurements within one scan cannot be obtained at the same time. Thus, if the micro-mobility is moving or the rider is rocking, mapping all LiDAR measurements within one scan onto $\Sigma_{w}$ using the self-pose information of the helmet calculated every scan period (100 ms) will result in distortion. To correct distortion in the LiDAR measurements, the self-pose of the helmet is estimated via extended Kalman filter (EKF) every 1.56 ms, in which the LiDAR measurements are obtained.

In this section, motion and observation models for EKF are discussed. As shown in Fig. 3, the translational velocities of the helmet on the *x*_*h*_, *y*_*h*_, and *z*_*h*_ axes in $\Sigma_{h}$ are expressed with *V*_*x*_, *V*_*y*_, and *V*_*z*_, respectively. The angular velocities (roll, pitch, and yaw angular velocities) of the helmet are expressed with $\dot{\phi }_{h}$, $\dot{\theta }_{h}$, and $\dot{\psi }_{h}$, respectively. For the motion model to correct the distortion in measurements from LiDARs mounted in vehicles (automobiles and two-wheel vehicles), our previous works [19, 20] considered only *V*_*x*_ as the translational velocity. The vehicle moves under the non-holonomic constraints. Thus, when the *x*-axis direction on the LiDAR coordinate system fixed at the LiDAR is consistent with the vehicle heading, it is sufficient to only consider *V*_*x*_ as the translational velocity.

However, in this paper, since the LiDAR is fixed at the helmet, it is very likely that the rider will move his/her head in arbitrary directions to check safety or due to being inattentive. In such cases, the *x*-axis direction of the helmet will not be consistent with the heading of the micro-mobility. Thus, velocities in all directions in $\Sigma_{h}$, ($V_{x} ,V_{y} ,V_{z}$), are considered.

Let us assume that the helmet moves at nearly constant translational and angular velocities within a very short time frame. The motion model of the helmet in the continuous time system is then expressed by

2 $$ \left( {\begin{array}{*{20}c} {\dot{x}} \\ {\dot{y}} \\ {\dot{z}} \\ {\dot{V}_{x} } \\ {\dot{V}_{y} } \\ {\dot{V}_{z} } \\ {\ddot{\phi }_{h} } \\ {\ddot{\theta }_{h} } \\ {\ddot{\psi }_{h} } \\ \end{array} } \right) = \left( {\begin{array}{*{20}c} \begin{gathered} V_{x} \cos \theta \cos \psi + V_{y} (\sin \phi \sin \theta \cos \psi - \cos \phi \sin \psi ) \hfill \\ + V_{z} (\cos \phi \sin \theta \cos \psi + \sin \phi \sin \psi ) \hfill \\ \end{gathered} \\ \begin{gathered} V_{x} \cos \theta \sin \psi + V_{y} (\sin \phi \sin \theta \sin \psi + \cos \phi \cos \psi ) \hfill \\ + V_{z} (\cos \phi \sin \theta \sin \psi - \sin \phi \cos \psi ) \hfill \\ \end{gathered} \\ { - V_{x} \sin \theta + V_{y} \sin \phi \cos \theta + V_{z} \cos \phi \cos \theta } \\ {w_{{\dot{V}_{x} }} } \\ {w_{{\dot{V}_{y} }} } \\ {w_{{\dot{V}_{z} }} } \\ {w_{{\ddot{\phi }_{h} }} } \\ {w_{{\ddot{\theta }_{h} }} } \\ {w_{{\ddot{\psi }_{h} }} } \\ \end{array} } \right) $$

where ($w_{{\dot{V}{}_{x}}}$,$w_{{\dot{V}{}_{y}}}$,$w_{{\dot{V}{}_{z}}}$) and ($w_{{\ddot{\phi }_{h} }}$,$w_{{\ddot{\theta }_{h} }}$,$w_{{\ddot{\psi }_{h} }}$) are the translational and angular acceleration disturbances, respectively.

The relationship between the angular velocities ${(}\dot{\phi }{,}\dot{\theta }{,}\dot{\psi }{)}$ of the helmet in $\Sigma_{w}$ and that $(\dot{\phi }_{h} {,}\dot{\theta }_{h} {,}\dot{\psi }_{h} )$ in $\Sigma_{h}$ is expressed as follows [26]:

3 $$ \left( {\begin{array}{*{20}c} {\dot{\phi }} \\ {\dot{\theta }} \\ {\dot{\psi }} \\ \end{array} } \right) = \left( {\begin{array}{*{20}c} 1 & {\tan \theta \sin \phi } & {\tan \theta \cos \phi } \\ 0 & {\cos \phi } & { - \sin \phi } \\ 0 & {\frac{\sin \phi }{{\cos \theta }}} & {\frac{\cos \phi }{{\cos \theta }}} \\ \end{array} } \right)\left( {\begin{array}{*{20}c} {\dot{\phi }_{h} } \\ {\dot{\theta }_{h} } \\ {\dot{\psi }_{h} } \\ \end{array} } \right) $$

where $$- 90^\circ < \theta < 90^\circ$$.

If Eq. (2) is discretized by considering Eq. (3) via Euler’s method, the helmet motion is modeled in the discrete time system by

4 $$ \left( {\begin{array}{*{20}c} {x\,(t + 1)} \\ {y\,(t + 1)} \\ {z\,(t + 1)} \\ {\phi \,(t + 1)} \\ {\theta \,(t + 1)} \\ {\psi \,(t + 1)} \\ \begin{gathered} V_{x} \,(t + 1) \hfill \\ V_{y} \,(t + 1) \hfill \\ V_{z} \,(t + 1) \hfill \\ \end{gathered} \\ {\dot{\phi }_{h} \,(t + 1)} \\ {\dot{\theta }_{h} \,(t + 1)} \\ {\dot{\psi }_{h} \,(t + 1)} \\ \end{array} } \right) = \left( {\begin{array}{*{20}l} {x(t)\, + \,a{}_{1}(t)\cos \theta (t)\cos \psi (t)\, + \,a_{2} (t)\left\{ {\sin \phi (t)\sin \theta (t)\cos \psi (t)\, - \,\cos \phi (t)\sin \psi (t)} \right\}\, + \,a_{3} (t)\left\{ {\cos \phi (t)\sin \theta (t)\cos \psi (t)\, + \,\sin \phi (t)\sin \psi (t)} \right\}} \hfill \\ {y(t)\, + \,a{}_{1}(t)\cos \theta (t)\sin \psi (t)\, + \,a_{2} (t)\left\{ {\sin \phi (t)\sin \theta (t)\sin \psi (t)\, + \,\cos \phi (t)\cos \psi (t)} \right\}\, + \,a_{3} (t)\left\{ {\cos \phi (t)\sin \theta (t)\sin \psi (t)\, - \,\sin \phi (t)\cos \psi (t)} \right\}} \hfill \\ {z(t)\, - \,a{}_{1}(t)\sin \theta (t)\, + \,a_{2} (t)\sin \phi (t)\cos \theta (t)\, + \,a_{3} (t)\cos \phi (t)\cos \theta (t)} \hfill \\ {\phi (t)\, + \,a{}_{4}(t)\, + \,\left\{ {a{}_{5}(t)\sin \phi (t)\, + \,a{}_{6}(t)\cos \phi (t)} \right\}\tan \theta (t)} \hfill \\ {\theta (t)\, + \,\left\{ {a{}_{5}(t)\cos \phi (t)\, - \,a{}_{6}(t)\sin \phi (t)} \right\}} \hfill \\ {\psi (t)\, + \,\left\{ {a{}_{5}(t)\sin \phi (t)\, + \,a{}_{6}(t)\cos \phi (t)} \right\}\frac{1}{\cos \theta (t)}} \hfill \\ \begin{gathered} V_{x} (t)\, + \,\tau w_{{\dot{V}x}} \hfill \\ V_{y} (t)\, + \,\tau w_{{\dot{V}y}} \hfill \\ V_{z} (t)\, + \,\tau w_{{\dot{V}z}} \hfill \\ \end{gathered} \hfill \\ {\dot{\phi }_{h} (t)\, + \,\tau w_{{\dot{\phi }h}} } \hfill \\ {\dot{\theta }_{h} (t)\, + \,\tau w_{{\ddot{\theta }h}} } \hfill \\ {\dot{\psi }_{h} (t)\, + \,\tau w_{{\ddot{\psi }h}} } \hfill \\ \end{array} } \right) $$

where *t* and *t* + 1 represent time steps, and τ is the sampling period. $$a_{1} = V_{x} \tau + \tau^{2} w_{{\dot{V}_{x} }} /2$$, $$a_{2} = V_{y} \tau + \tau^{2} w_{{\dot{V}y}} /2$$, $$a_{3} = V_{z} \tau + \tau^{2} w_{{\dot{V}_{z} }} /2$$, $$a_{4} = \dot{\phi }_{h} \tau + \tau^{2} w_{{\ddot{\phi }_{h} }} /2$$, $$a_{5} = \dot{\theta }_{h} \tau + \tau^{2} w_{{\ddot{\theta }_{h} }} /2$$, and $$a_{6} = \dot{\psi }_{h} \tau +$$$$\tau^{2} w_{{\ddot{\psi }_{h} }} /2$$.

Equation (4) is expressed as a vector form:

5 $$ {{\varvec{\upxi}}}(t + 1) = {\mathbf{f}}\left[ {{{\varvec{\upxi}}}(t),{\mathbf{w}},\tau } \right] $$

where $${{\varvec{\upxi}}} = (x,y,z,\phi ,\theta ,\psi ,V_{x} ,V_{y} ,V_{z} ,\dot{\phi }_{h} ,\dot{\theta }_{h} ,\dot{\psi }_{h} )^{T}$$ and $${\mathbf{w}} = (w_{{\dot{V}x}} ,$$ $$w_{{\dot{V}y}} ,w_{{\dot{V}z}} ,w_{{\ddot{\phi }_{h} }} ,w_{{\ddot{\theta }_{h} }} ,w_{{\ddot{\psi }_{h} }} )^{T}$$.

The IMU observation period (10 ms) is expressed as $\tau_{IMU}$. The information on the attitude angle (roll and pitch angles) and attitude angular velocity (roll, pitch, and yaw angular velocities) of the helmet obtained at time $t\tau_{IMU}$ with IMU is denoted as ${\mathbf{z}}_{IMU} (t)$. The observation equation is

6 $$ {\mathbf{z}}_{IMU} (t) = {\mathbf{H}}_{IMU} \xi (t) + \Delta {\mathbf{z}}_{IMU} (t), $$

where $\Delta {\mathbf{z}}_{IMU}$ is the observation noise, and ${\mathbf{H}}_{IMU}$ is the observation matrix expressed as

$$ {\mathbf{H}}_{IMU} = \left( {\begin{array}{*{20}c} 0 & 0 & 0 & 1 & 0 & 0 & 0 & 0 & 0 & 0 & 0 & 0 \\ 0 & 0 & 0 & 0 & 1 & 0 & 0 & 0 & 0 & 0 & 0 & 0 \\ 0 & 0 & 0 & 0 & 0 & 0 & 0 & 0 & 0 & 1 & 0 & 0 \\ 0 & 0 & 0 & 0 & 0 & 0 & 0 & 0 & 0 & 0 & 1 & 0 \\ 0 & 0 & 0 & 0 & 0 & 0 & 0 & 0 & 0 & 0 & 0 & 1 \\ \end{array} } \right) $$

If the self-pose of the helmet obtained at time $t\tau$ with NDT SLAM is denoted as ${\mathbf{z}}_{NDT} (t)$, the observation equation is

7 $$ {\mathbf{z}}_{NDT} (t) = {\mathbf{H}}_{NDT} \xi (t) + \Delta {\mathbf{z}}_{NDT} (t), $$

where $\Delta {\mathbf{z}}_{NDT}$ observation noise, and the observation matrix ${\mathbf{H}}_{NDT}$ is expressed as

$$ {\mathbf{H}}_{NDT} = \left( {\begin{array}{*{20}c} 1 & 0 & 0 & 0 & 0 & 0 & 0 & 0 & 0 & 0 & 0 & 0 \\ 0 & 1 & 0 & 0 & 0 & 0 & 0 & 0 & 0 & 0 & 0 & 0 \\ 0 & 0 & 1 & 0 & 0 & 0 & 0 & 0 & 0 & 0 & 0 & 0 \\ 0 & 0 & 0 & 1 & 0 & 0 & 0 & 0 & 0 & 0 & 0 & 0 \\ 0 & 0 & 0 & 0 & 1 & 0 & 0 & 0 & 0 & 0 & 0 & 0 \\ 0 & 0 & 0 & 0 & 0 & 1 & 0 & 0 & 0 & 0 & 0 & 0 \\ \end{array} } \right) $$

### Distortion correction

**Fig. 4:**
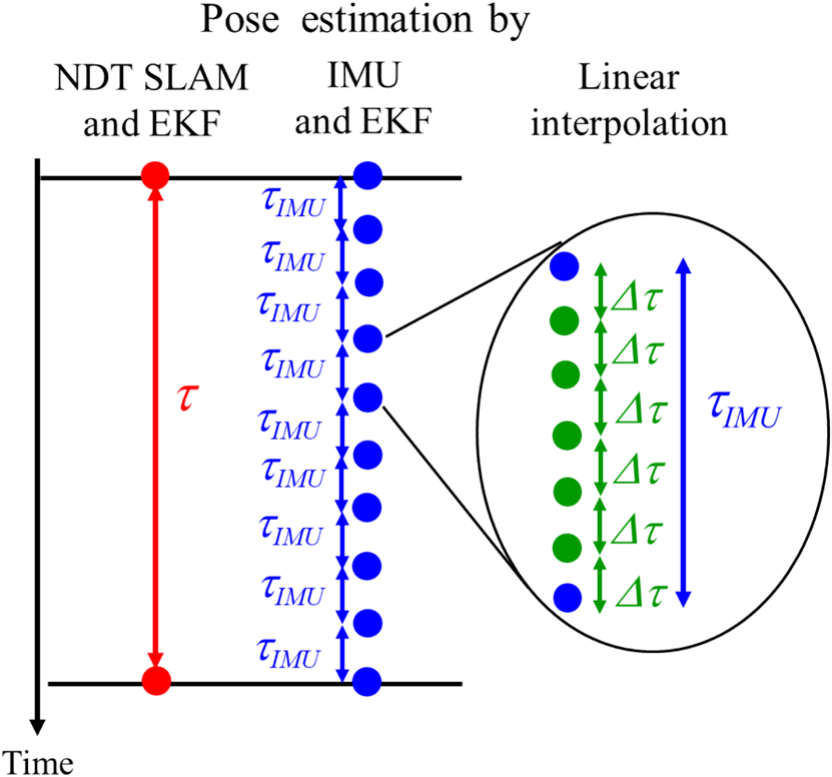
Flow of pose estimation for distortion correction of LiDAR measurements. $\tau$ is the LiDAR scan period (100 ms), $\tau_{IMU}$ is the IMU observation period (10 ms), and $\Delta \tau$ is the observation period of LiDAR measurements (1.56 ms)

Figure 4 shows the flow of the pose estimation for the distortion correction of LiDAR measurements. The self-pose of the helmet is calculated using NDT SLAM every 100 ms (the LiDAR scan period). Since the IMU observation period is 10 ms, the attitude angle and angular velocity of the helmet are obtained ten times from IMU during the LiDAR scan period. Furthermore, since the observation period of the LiDAR measurements is 1.56 ms, LiDAR measurements are obtained six times during the IMU observation period.

Therefore, the self-pose of the helmet is estimated every 100 ms using NDT SLAM and EKF, in which the motion and observation models (Eqs. (5) and (7)) are utilized. Furthermore, it is estimated every 10 ms using IMU and EKF, in which the motion and observation models (Eqs. (5) and (6)) are utilized. The self-pose of the helmet every 1.56 ms, is obtained by linearly interpolating the information of the self-pose estimated every 10 ms.

The distortion in LiDAR measurements is corrected using the information of the self-pose estimated every 1.56 ms. The details of the correction algorithm [20] are described in the appendix.

## Extraction of LiDAR measurements relating to stationary objects

### Extraction of object measurement

In a dynamic environment with moving objects, such as cars, two-wheeled vehicles, and pedestrians, LiDAR measurements relating to stationary objects must be extracted from all LiDAR measurements within one scan to build an environmental map. To that end, distortion-corrected LiDAR measurements are divided into measurements from stationary objects (referred to as stationary measurements) and those from moving objects (moving measurements) by the following method.

**Fig. 5:**
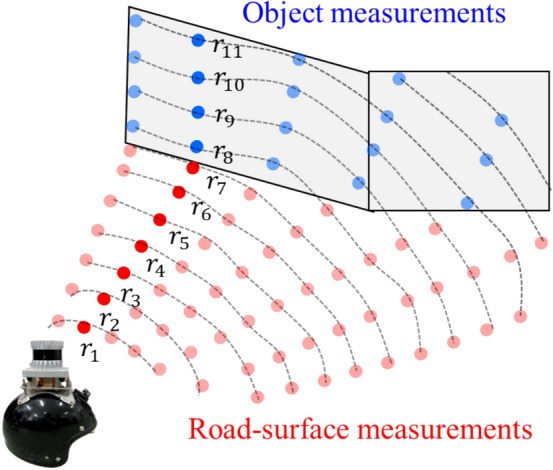
Extraction of LiDAR measurements relating to objects. The red and blue points indicate the LiDAR measurements relating to the road surface and object, respectively

First, distortion-corrected LiDAR measurements are sorted into road surface measurements taken from road surfaces and object measurements taken from 3D objects, such as buildings, trees and automobiles. As shown in Fig. 5, the LiDAR measurements obtained in any horizontal direction are numbered *r*_1_, *r*_2_,..., *r*_*n*_ in order from the measurement closest to the helmet.

Next, the angle $\alpha$ of the line connecting two points (*r*_1_ and *r*_2_) relative to the *xy* plane in $\Sigma_{w}$ is obtained. If $\alpha \le$ 10°, *r*_2_ is considered the road surface measurement. This process is performed sequentially on other measurements *r*_3_,..., *r*_*n*_, and measurements with $\alpha \, > \,10^{ \circ }$, such as *r*_8_–*r*_11_ in Fig. 5, are considered the object measurements. This process is performed on all LiDAR measurements, and only the object measurements are used for subsequent processes.

When the helmet is tilted significantly up and down (in pitch direction) or left and right (in roll direction), laser beams emitted from the LiDAR would hit the body of the micro-mobility. Therefore, LiDAR measurements obtained within a radius of 1 m from the helmet in $\Sigma_{w}$ are not used to determine road surface and object measurements. Furthermore, when the helmet is tilted significantly, LiDAR laser beams might not hit road surfaces but hit only 3D objects. However, even in this case, the object measurements can be extracted because the angle $\alpha$ is estimated in $\Sigma_{w}$.

As mentioned above, the angle threshold to classify road surface and object measurements is set to 10°. If it is small, road slopes will be mis-detected as 3D objects. Furthermore, since the self-pose information of the helmet using NDT SLAM is needed to estimate the angle $\alpha$ in $\Sigma_{w}$, the error in the self-pose estimate would cause the mis-detection of 3D objects. In general, the slope of steep roads for vehicles is about 6°. Thus, in this paper, the threshold value of 10°, which is larger than 6°, is set.

### Extraction of stationary object measurement

The object measurements are mapped on the grid map (elevation map) expressed in $\Sigma_{w}$. The cell size of the grid map is a square with a side of 0.3 m. The time to occupy the same cell is short for moving measurements and long for stationary measurements. Thus, using the occupancy grid method based on the cell-occupation time [27], cells are classified into cells occupied by moving measurements (referred to as moving cells) and those occupied by stationary measurements (stationary cells). In other words, cells with an occupation time of 0.8 s or more are determined to be stationary cells, while cells with an occupation time of less than 0.8 s are determined to be moving cells. Here, cell-occupation time is not measured for cells occluded behind objects because these are invisible by the LiDAR.

Since object measurements occupy multiple cells, adjacent occupied cells are clustered. When the ratio of stationary cells exceeds 50% in a cluster, the cluster is considered to be a stationary cell cluster. Only the LiDAR measurements within the stationary cell cluster are used to build an environmental map.

In our preliminary experiments, LiDAR was able to correctly detect objects located within the range of about 50 m from the helmet. Thus, we set up a grid map in ± 35 m squares from the helmet; the distance from the helmet to the vertex of the square is about 50 m. Considering the resolution (0.35°) of the horizontal viewing angle of LiDAR, the cell size of the grid map is set to 0.3 m so that at least one measurement of LiDAR could be occupied in a cell 50 m away from the helmet. Assuming that the width and length of a pedestrian are 0.4 m, and the walking speed is more than 1 m/s, the time that the LiDAR measurements related to the pedestrian occupy a cell is less than 0.8 s. Therefore, the threshold of the occupation time to determine stationary and moving cells is set to 0.8 s.

## Fundamental experiments

**Fig. 6:**
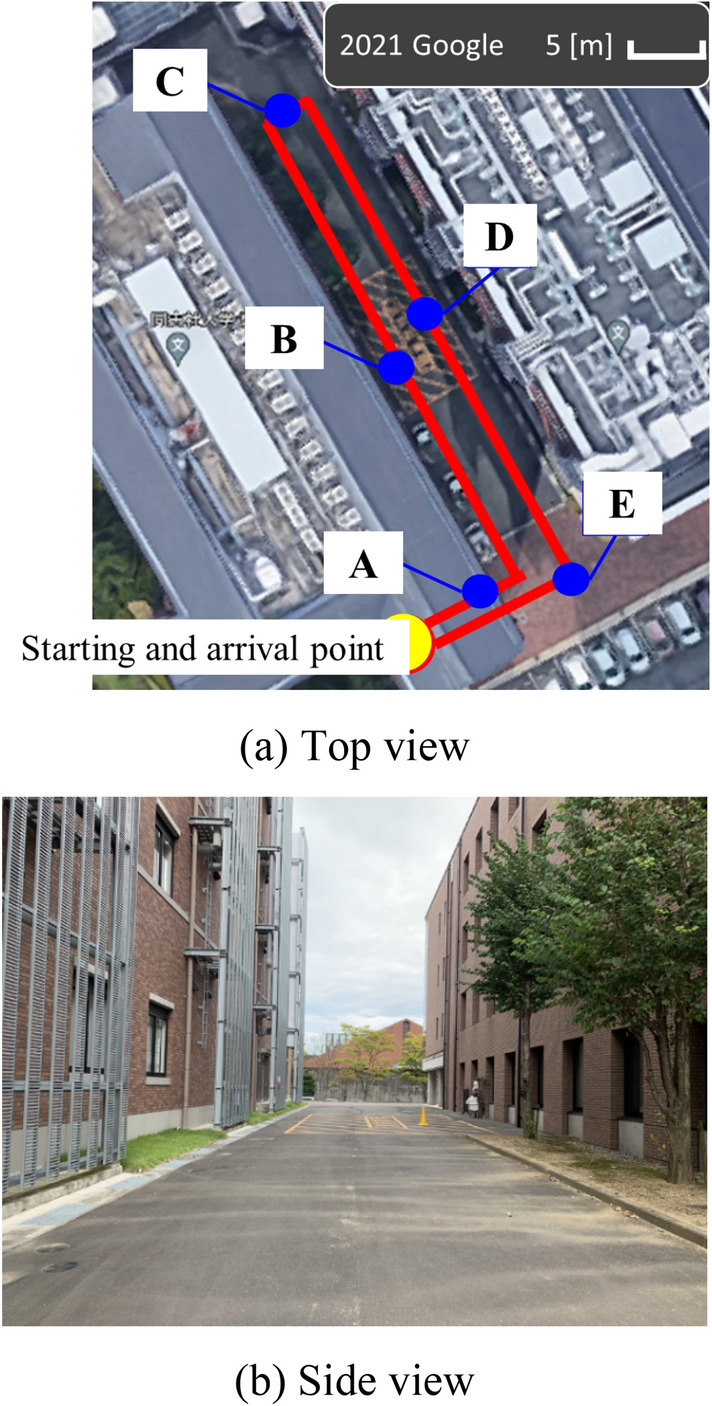
Experimental environment. In ** a** the red line indicates the movement path of micromibility.** a **Top view.** b** Side view

First, to examine the effectiveness of the proposed motion model (the motion model without non-holonomic constraints), an environmental map is built by driving the micromobility (Segway PT × 2 SE) in an environment shown in Fig. 6. The traveled distance of the micro-mobility is about 120 m, and a maximum velocity is about 11 km/h.

**Fig. 7:**
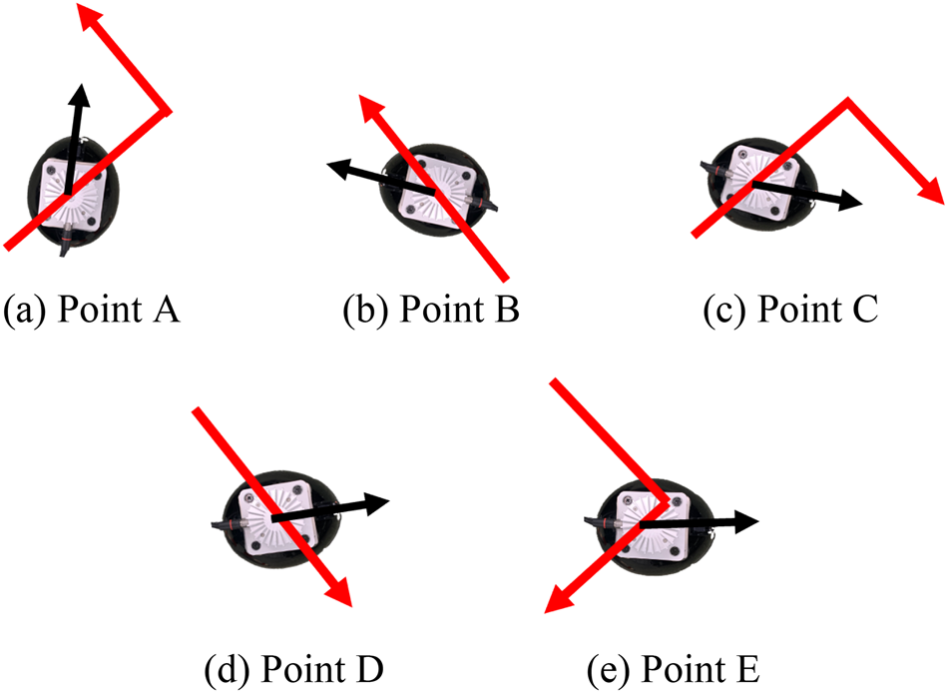
Moving direction (red) of micromobility and heading direction (black) of rider (top view)** a** Point A.** b** Point B.** c** Point C.** d** Point D.** e**Point E

At points A–E in Fig. 6 (a), the following actions are performed, in which the *x*_*h*_-axis direction in *Σ*_*h*_ (the heading direction of the rider) is not consistent with the moving direction of the micromobility (see Fig. 7).

Point A: Turning left while visually confirming safety to the left.

Point B: Avoiding a left obstacle while confirming the object visually.

Point C: Making a U-turn while confirming the safety of the direction visually.

Point D: Driving forward while inattentively glancing to the left.

Point E: Turning right while visually confirming safety to the left.

**Fig. 8:**
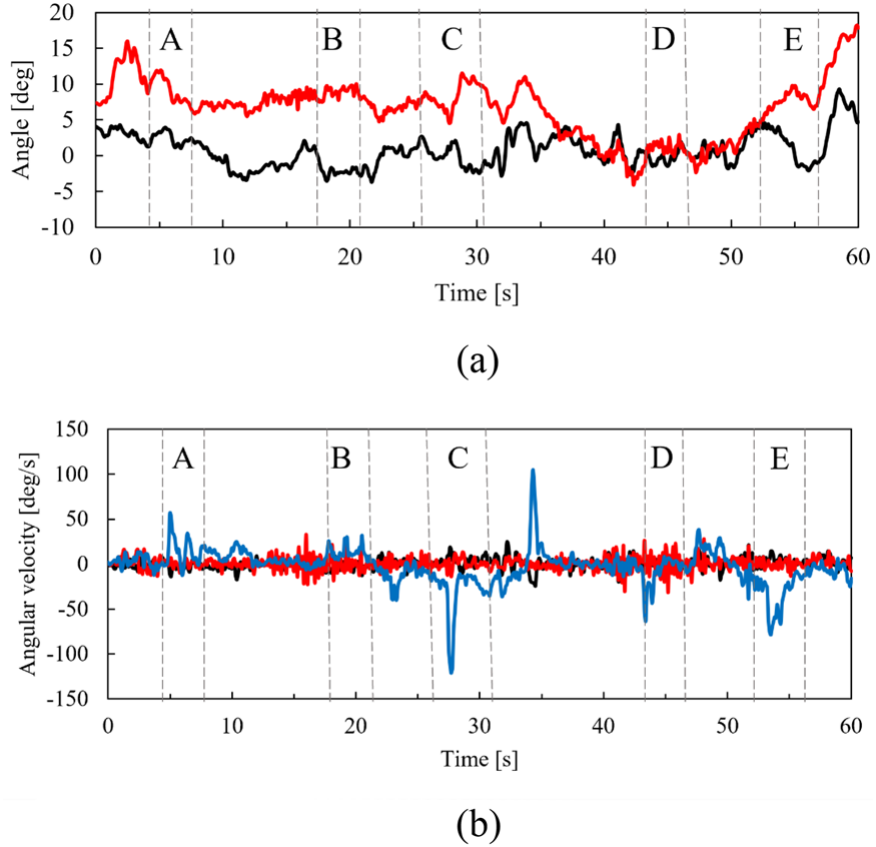
Attitude angle and angular velocity of helmet.** a** Roll (balck) and pitch (red) angles.** b** Roll (black), pitch (red), and yaw (blue) angular velocities

IMU output (attitude angle and angular velocity of the helmet) during driving is shown in Fig. 8. The locations indicated by A–E in the figure indicate the time during which the micro-mobility travels points A–E. Although the helmet was tilted up and down (− 4.1° to 18.2° in the pitch direction) and left and right (− 3.7 to 9.3° in the roll direction), LiDAR was able to correctly obtain object measurements.

**Fig. 9:**
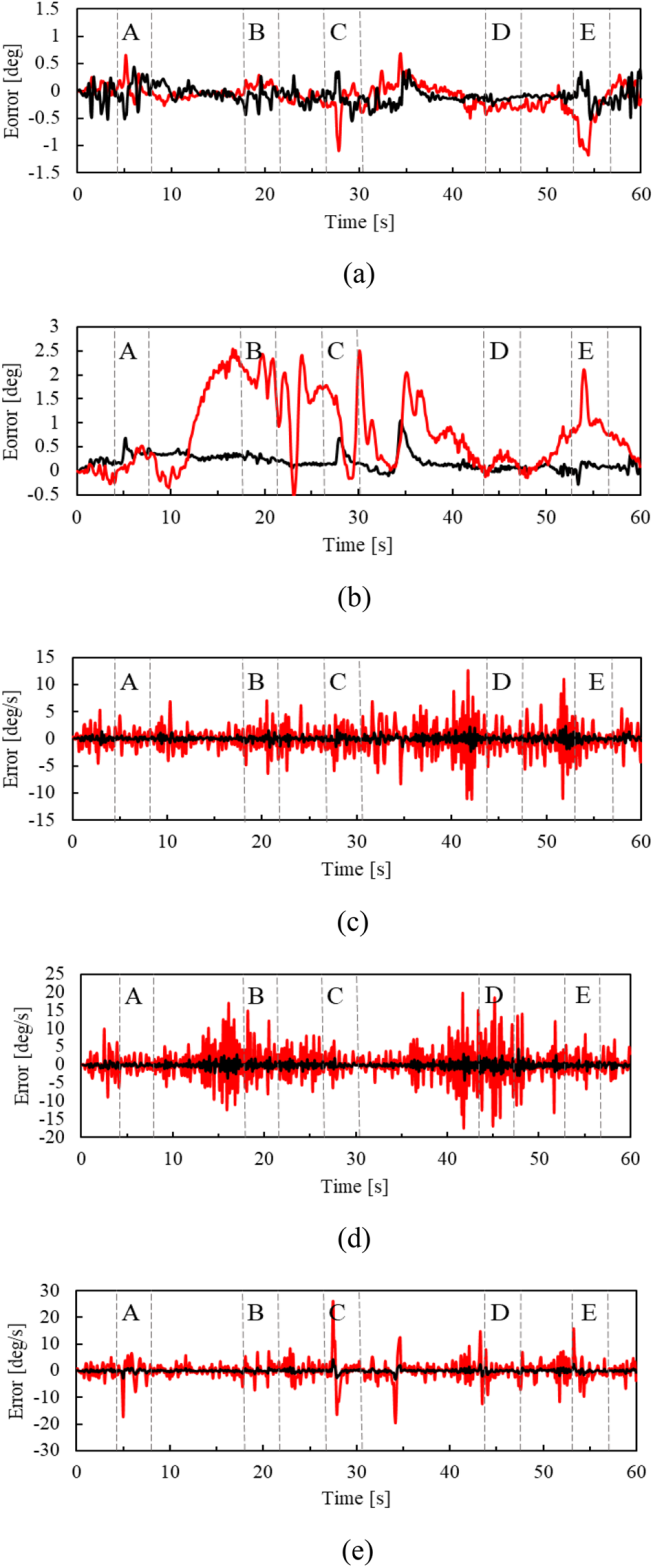
Estimate error in attitude angle and angular velocity of helmet. The black and red lines indicate the errors by the proposed and conventional motion models, respectively.** a** Roll angle.** b** Pitch angle.** c** Roll angular velocity.** d** Pitch angular velocity.** e** Yaw angular velocity.

Figure 9 shows the error in the attitude angle (roll and pitch angles) and angular velocity (roll, pitch, and yaw angular velocities) of the helmet estimated by EKF during the experiment. Here, for convenience, the IMU output (attitude angle and angular velocity) is considered the true value. For comparison, the estimation error for the conventional motion model (the motion model with non-holonomic constraints) [20] is also shown in the figure.

It is clear from these figures that the estimation error in the attitude angle and angular velocity by the proposed motion model is smaller than that by the conventional motion model.

**Fig. 10:**
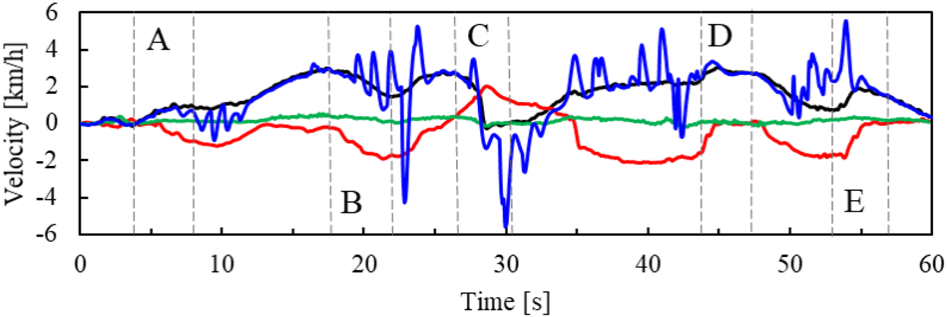
Velocity estimate of helmet. The black, red, green lines indicate the velocity in the x_h_, y_h_, and z_h_ axes directions, respectively, estimated by the proposed motion model. The blue line indicates the velocity estimated by the conventional motion model

Figure 10 shows the translational velocity of the helmet estimated by the proposed motion model and the conventional motion model. The velocity estimated by the conventional motion model fluctuates much more than that by the proposed motion model.

**Fig. 11:**
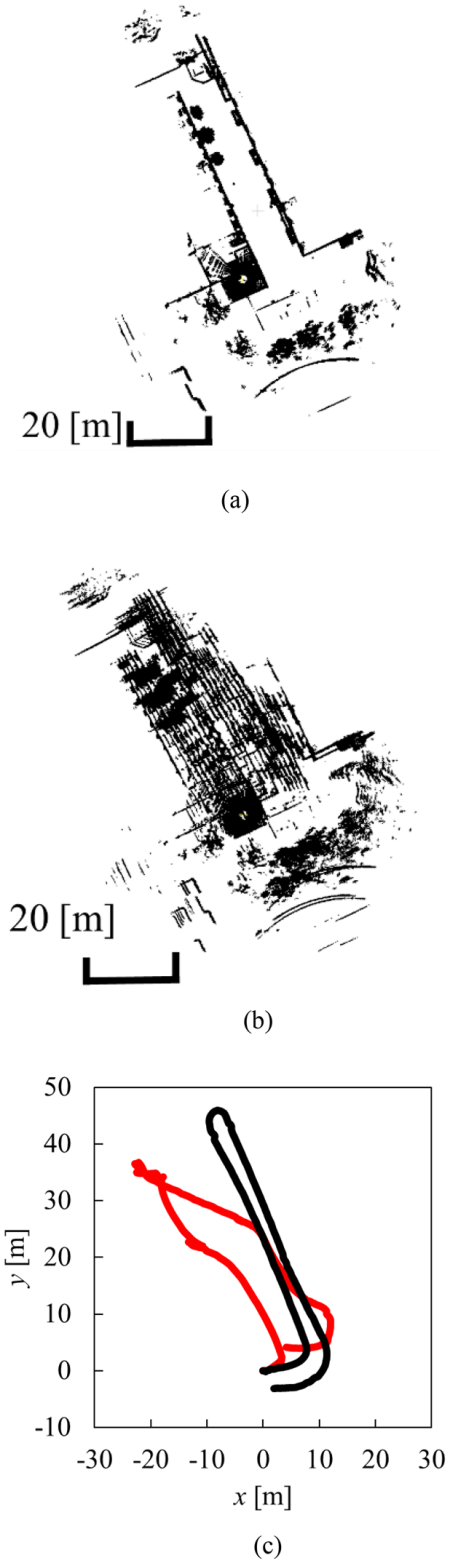
SLAM result (top view).** a** Environment map by the proposed motion model.** b** Environmental map by the conventional motion model. ** c** position of helmet The black and red lines in indicate the results by the proposed and conventional motion models, respectively

Figure 11 shows the result of the environmental map building and self-position estimate obtained by NDT SLAM. In comparison with the photo of the environment and movement path of the micro-mobility in Fig. 6, SLAM result by the proposed motion model is more accurate than that by the conventional motion model.

**Fig. 12:**
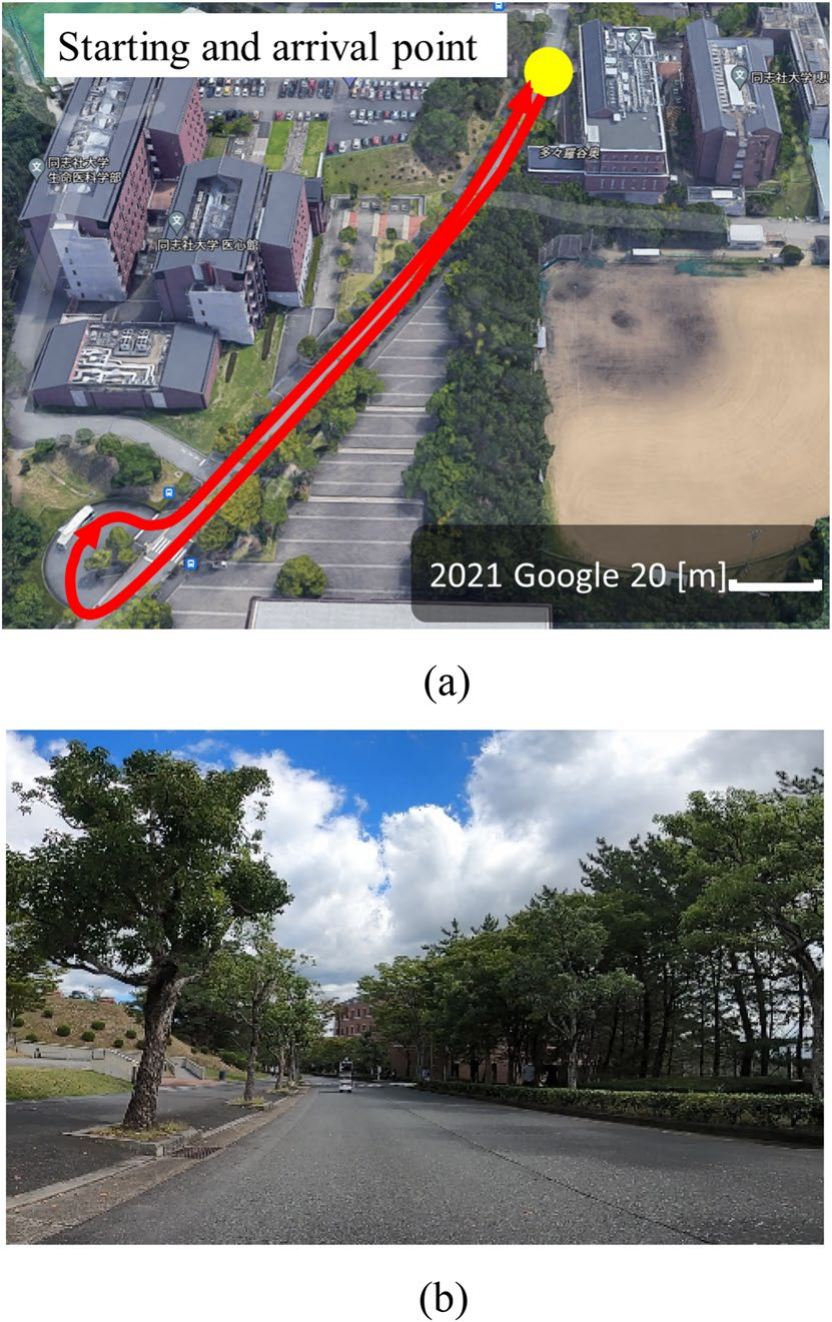
Experimental environment.** a** Top view.** b** Side view

**Fig. 13:**
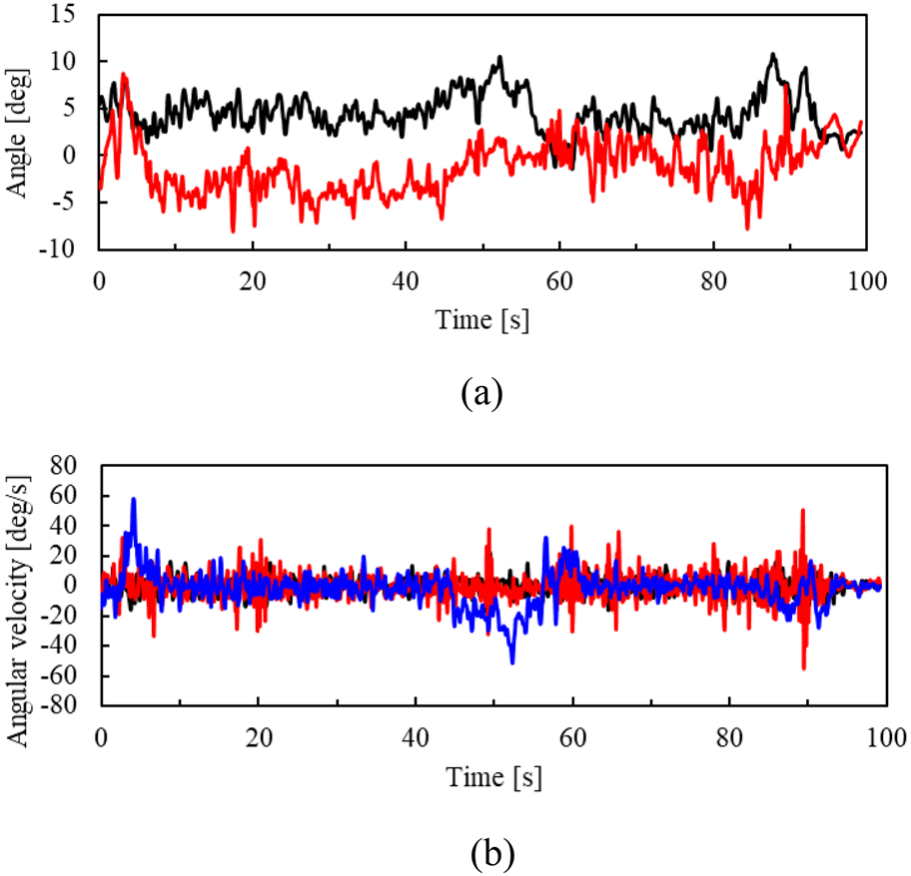
Attitude angle and angular velocity of helmet.** a** Roll (black) and pitch (red) angles.** b** Roll (black), pitch (red), and yaw (blue) angular velocities

Next, to quantitatively evaluate the performance of NDT SLAM, another experiment is conducted in the environment shown in Fig. 12 by riding a bicycle (micro-mobility) and driving a long distance (about 500 m) at a high velocity (about 30 km/h). IMU output (attitude angle and angular velocity of the helmet) during driving is shown in Fig. 13. Although the helmet was tilted up and down (− 9.2–9.8° in the pitch direction) and left and right (− 1.8–10.6° in the roll direction), LiDAR was able to correctly obtain object measurements.

The NDT SLAM performance is examined under the following four conditions:

Condition 1: SLAM with the proposed motion model and distortion correction method (the proposed method).

Condition 2: SLAM with the proposed motion model and without distortion correction method.

Condition 3: SLAM with the conventional motion model and distortion correction method.

Condition 4: SLAM with the conventional motion model and without distortion correction method.

**Fig. 14:**
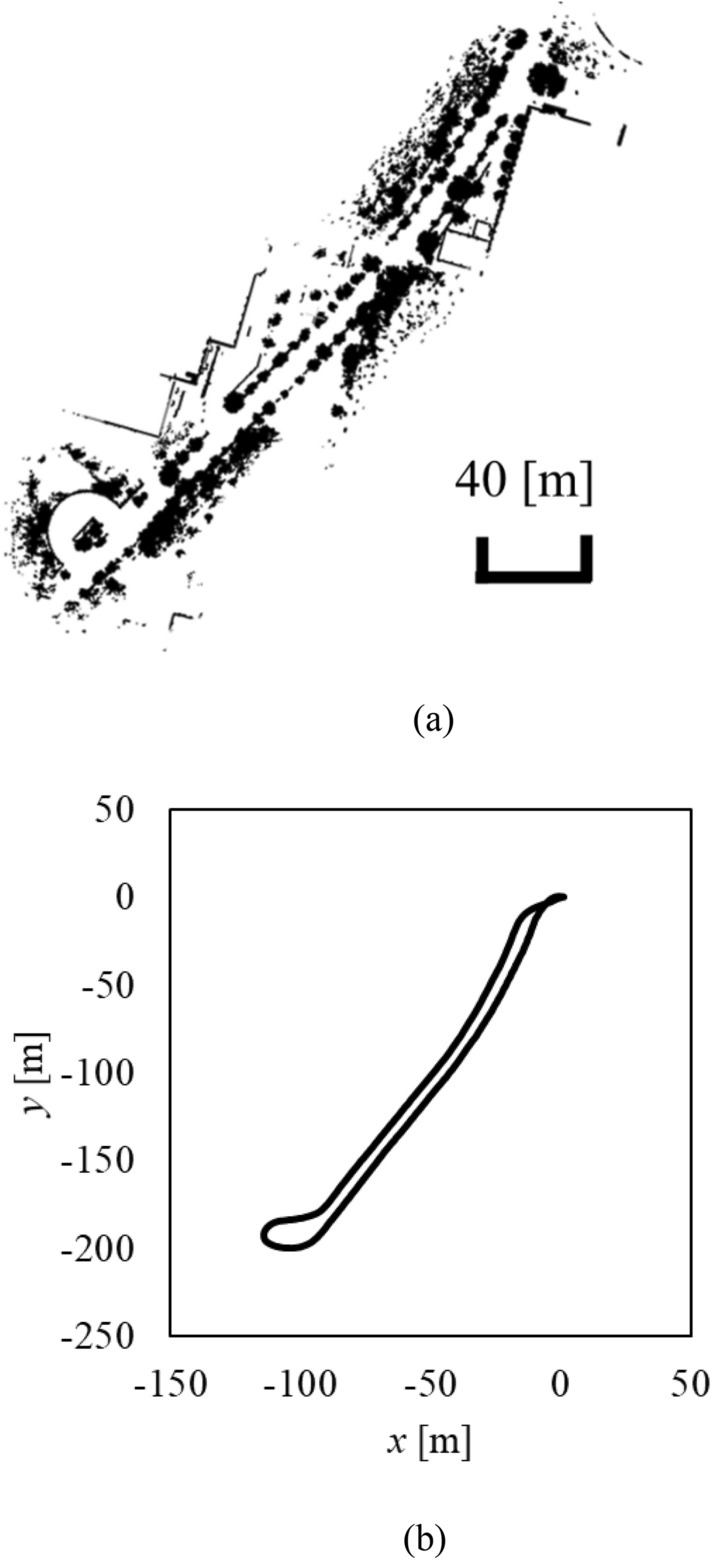
SLAM result in condition 1 (top view).** a** Environmental map.** b** Position estimate of helmet

As an example of SLAM results, the result for Condition 1 is shown in Fig. 14.

In SLAM, the mapping performance is equivalent to the localization performance. Therefore, to quantitatively evaluate the mapping performance, the self-position of the helmet at the arrival point is obtained using the following steps:

Step (1) A positioning unit (PU) consisting of a LiDAR (Velodyne 32 layer LiDAR, HDL-32E) and global navigation satellite system/inertial navigation system (GNSS/INS, Novatel PwrPak7-E1) is placed at the starting and arriving points in Fig. 12(a) to acquire the LiDAR measurements and PU pose.

Step (2) When the micro-mobility starts, NDT scan matching is performed using the measurements of the helmet-mounted LiDAR and those of the PU LiDAR to obtain the pose of the helmet relative to the PU. Then, using the relative pose and PU pose in $\Sigma_{gnss}$, the self-pose of the helmet at the starting point in $\Sigma_{gnss}$ is calculated. Here, $\Sigma_{gnss}$ is defined, in which the origin is the PU position calculated by the GNSS/INS and *x*, *y*, and *z* axes are the east/west, north/south, and zenith directions, respectively.

Step (3) Using the self-pose acquired in Step (2) as the initial pose of the helmet, NDT SLAM is performed with the helmet-mounted LiDAR.

Step (4) When the micro-mobility arrives at the arrival point, NDT scan matching is performed using the measurements of the helmet-mounted LiDAR and those of PU LiDAR to calculate the self-position of the helmet at the arrival point in $\Sigma_{gnss}$.

Step (5) The self-position of the helmet obtained at the arrival point in Step 4) is taken as the true position, and the that obtained at the arrival point by NDT SLAM is taken as the estimate. Then, the error $(\Delta x,\Delta y,\Delta z)$ in the self-position estimation is calculated.

Table 1 shows the estimate error $\sqrt {\Delta x^{2} + \Delta y^{2} + \Delta z^{2} }$ of the self-position. These are the results of three runs of the micro-mobility. In the three runs, there were a total of 111 moving objects (106 pedestrians and five cars). It is clear from the table that the proposed method (Condition 1) performs SLAM more accurately than the other methods (Conditions 2, 3, and 4).

**Table 1:** Error of position estimate of helmet at arrival point (m)

	Condition 1	Condition 2	Condition 3	Condition 4
Run 1	0.23	5.91	12.99	12.71
Run 2	1.93	15.10	15.44	57.73
Run 3	0.71	6.03	24.98	6.71

## Conclusions

This paper presented a method to build an environmental map (3D point-cloud map) through NDT SLAM using a LiDAR mounted on the rider helmet of a micro-mobility.

The motion of the helmet was represented by a 3D motion model without non-holonomic constraints. Then, the 3D self-pose (position and attitude angle) of the helmet was estimated at the observation period of LiDAR measurements via EKF based on the self-pose of the helmet obtained by NDT SLAM and IMU information. Based on the estimated self-pose, the distortion in the LiDAR measurements, which was caused by the motion of the micro-mobility and rider, was corrected. Furthermore, the LiDAR measurements relating to stationary objects were extracted from the distortion-corrected LiDAR measurements, and an environmental map was built using NDT SLAM. The effectiveness of the presented method was validated through the experiments on road environments on the university campus.

In future works, the accuracy of environmental maps will be improved using Graph SLAM and publicity available maps, such as OpenStreetMap and PLATEAU. In addition, instead of a mechanical LiDAR, a small size and light-weight solid-state LiDAR will be mounted on a rider helmet.

## Data Availability

The data that support the findings of this study are available from the corresponding author upon reasonable request.

## Appendix: Algorithm of distortion correction

The LiDAR scan period (100 ms), IMU observation period (10 ms), and observation period of LiDAR measurements (1.56 ms) are expressed as $\tau$, $\tau_{IMU}$, and $\Delta \tau$, respectively. The relationships of these periods are then $\tau = 10\tau_{IMU}$ and $\tau_{IMU} \simeq 6\Delta \tau$.

The distortion in the LiDAR measurements obtained between times $(t - 1)\tau + \Delta \tau$ and $(t - 1)\tau + 64\Delta \tau$ (i.e., $(t - 1)\tau + \Delta \tau$ and $t\tau$) can be corrected in the following six steps:

Step (1) The state estimate at time $(t - 1)\tau + (k - 1)\tau_{IMU}$, where *k* = 1, 2,$\cdots$, 11, is expressed as ${\hat{\mathbf{\xi }}}^{(k - 1)} (t - 1)$, and its error covariance as ${{\varvec{\Gamma}}}^{(k - 1)} (t - 1)$. The prediction algorithm of EKF obtains the state prediction ${\hat{\mathbf{\xi }}}^{(k/k - 1)} (t - 1)$ and its error covariance ${{\varvec{\Gamma}}}^{(k/k - 1)} (t - 1)$ at $(t - 1)\tau$ + $k\tau_{IMU}$ by

8 $$ \left. {\frac{{\widehat{\xi }_{t - 1}^{{{\raise0.7ex\hbox{$k$} \!\mathord{\left/ {\vphantom {k {k - 1}}}\right.\kern-0pt} \!\lower0.7ex\hbox{${k - 1}$}}}} \,\, = \,\,f\widehat{\xi }_{t - 1}^{k - 1} ,\,0,\,\tau_{IMU} }}{{\Gamma_{t - 1}^{{{\raise0.7ex\hbox{$k$} \!\mathord{\left/ {\vphantom {k {k - 1}}}\right.\kern-0pt} \!\lower0.7ex\hbox{${k - 1}$}}}} \,\, = \,\,F_{t - 1} \Gamma_{t - 1}^{k - 1} F_{t - 1}^{T} \, + \,G_{{\left( {t - 1} \right)}} \,QG\frac{T}{t - 1}}}} \right\} $$

where ***F*** = $\partial {\mathbf{f}}/\partial {\hat{\mathbf{\xi }}}$, ***G*** = $\partial {\mathbf{f}}/\partial {\mathbf{w}}$. ***Q*** is the covariance matrix of the plant noise ***w***. In the experiment in Sect. 6, an identity matrix is set for ***Q***.

Step (2) At $(t - 1)\tau + k\tau_{IMU}$, the attitude angle and angular velocity ${\mathbf{z}}_{IMU}$ of the helmet is obtained from IMU. The observation update algorithm of EKF can then calculate the state estimate ${\hat{\mathbf{\xi }}}^{(k)} (t - 1)$ and its error covariance ${{\varvec{\Gamma}}}^{(k)} (t - 1)$ as follows:

9 $$ \left. {\begin{array}{*{20}l} {{\hat{\mathbf{\xi }}}^{(k)} (t - 1) = {\hat{\mathbf{\xi }}}^{(k/k - 1)} (t - 1) + {\mathbf{K}}\{ {\mathbf{z}}_{IMU} - {\mathbf{H}}_{IMU} {\hat{\mathbf{\xi }}}^{(k/k - 1)} (t - 1)\} } \hfill \\ {{{\varvec{\Gamma}}}^{(k)} (t - 1) = {{\varvec{\Gamma}}}^{(k/k - 1)} (t - 1) - {\mathbf{KH}}_{IMU} {{\varvec{\Gamma}}}^{(k/k - 1)} (t - 1)} \hfill \\ \end{array} } \right\} $$

where the Kalman gain ${\mathbf{K}}(t)$ and observation prediction error ${\mathbf{S}}(t)$ are given by

10 $$ \left. \begin{gathered} {\mathbf{K}}(t) = {{\varvec{\Gamma}}}^{(k/k - 1)} (t - 1){\mathbf{H}}_{IMU}^{T} {\mathbf{S}}^{ - 1} (t - 1) \hfill \\ {\mathbf{S}}(t) = {\mathbf{H}}_{IMU}^{{}} {{\varvec{\Gamma}}}^{(k/k - 1)} (t - 1){\mathbf{H}}_{IMU}^{T} + {\mathbf{R}}_{IMU}^{{}} \hfill \\ \end{gathered} \right\} $$

where $${\mathbf{R}}_{IMU}^{{}}$$ is the covariance of the sensor noise $$\Delta {\mathbf{z}}_{IMU}$$. In the experiment in Sect. 6, $${\mathbf{R}}_{IMU}^{{}} = {\text{diag}} [(0.015)^{2} ,(0.015)^{2} ,$$ $$(0.05)^{2} ,(0.05)^{2} ,$$$$(10)^{2} ]$$ is used.

Among the state estimate ${\hat{\mathbf{\xi }}}^{(k)} (t - 1)$, the state estimate related to the self-pose of the helmet, $(x,y,z,\phi ,\theta ,\psi )$, is expressed as ${\hat{\mathbf{X}}}^{(k)} (t - 1)$.

Step (3) Using the self-poses of the helmet, $${\hat{\mathbf{X}}}^{(k - 1)} (t - 1)$$ and $${\hat{\mathbf{X}}}^{(k)} (t - 1)$$, estimated at times $$(t - 1)\tau +$$$$(k - 1)\tau_{IMU}$$ and $$(t - 1)\tau$$ + $$k\tau_{IMU}$$, respectively, the self-pose $${\hat{\mathbf{X}}}^{(k - 1)} (t - 1,j)$$ at time $$(t - 1)\tau + (k - 1)\tau_{IMU} + j\Delta \tau$$ (where *j* = 1, 2,...,5) is linearly interpolated by the following equation:

11 $$ {\hat{\mathbf{X}}}^{(k - 1)} (t - 1,j) = {\hat{\mathbf{X}}}^{(k - 1)} (t - 1) + \frac{{{\hat{\mathbf{X}}}^{(k)} (t - 1) - {\hat{\mathbf{X}}}^{(k - 1)} (t - 1)}}{{\tau_{IMU} }}j\Delta \tau $$

Step (4) Using ${\hat{\mathbf{X}}}^{(k - 1)} (t - 1,j)$ and Eq. (1), the 3D positions ${\mathbf{p}}_{hi}^{(k - 1)} (t - 1,j)$ of the LiDAR measurements obtained in $\Sigma_{h}$ at $(t - 1)\tau + (k - 1)\tau_{IMU} + j\Delta \tau$ is transformed into the 3D positions ${\mathbf{p}}_{i}^{(k - 1)} (t - 1,j)$ in $\Sigma_{w}$ as follows:

12 $$ \left( {\begin{array}{*{20}c} {{\mathbf{p}}_{i}^{(k - 1)} (t - 1,j)} \\ 1 \\ \end{array} } \right) = {\mathbf{\rm T}}({\hat{\mathbf{X}}}^{(k - 1)} (t - 1,j))\left( {\begin{array}{*{20}c} {{\mathbf{p}}_{hi}^{(k - 1)} (t - 1,j)} \\ 1 \\ \end{array} } \right) $$

Step (5) Using the self-pose estimate ${\hat{\mathbf{X}}}^{(10)} (t - 1)$ of the helmet at time $t\tau$, the LiDAR measurements ${\mathbf{p}}_{i}^{(k - 1)} (t - 1,j)$ are again transformed to ${\mathbf{p}}_{hi}^{*} (t)$ in $\Sigma_{h}$ at time $t\tau$ by

13 $$ \left( {\begin{array}{*{20}c} {{\mathbf{p}}_{hi}^{*} (t)} \\ 1 \\ \end{array} } \right) = {\mathbf{\rm T}}({\hat{\mathbf{X}}}^{(10)} (t - 1))^{ - 1} \left( {\begin{array}{*{20}c} {{\mathbf{p}}_{i}^{(k - 1)} (t - 1,j)} \\ 1 \\ \end{array} } \right) $$

With the above equation, the 3D positions of the LiDAR measurements obtained between times $(t - 1)\tau + \Delta \tau$ and $t\tau$ can be corrected.

Step (6) Using the distortion-corrected LiDAR measurements, the self-pose ${\mathbf{z}}_{NDT}$ of the helmet at time $t\tau$ is calculated via NDT SLAM.

The observation update algorithm of the EKF obtains the state estimate ${\hat{\mathbf{\xi }}}(t)$ and its error covariance ${{\varvec{\Gamma}}}(t)$ at time $t\tau$ as follows:

14 $$ \left. {\begin{array}{*{20}l} {{\hat{\mathbf{\xi }}}(t) = {\hat{\mathbf{\xi }}}^{(10)} (t - 1) + {\mathbf{K}}(t)\{ {\mathbf{z}}_{NDT} (t) - {\mathbf{H}}_{NDT} {\hat{\mathbf{\xi }}}^{(10)} (t - 1)\} } \hfill \\ {{{\varvec{\Gamma}}}(t) = {{\varvec{\Gamma}}}^{(10)} (t - 1) - {\mathbf{K}}(t){\mathbf{H}}_{NDT} {{\varvec{\Gamma}}}^{(10)} (t - 1)} \hfill \\ \end{array} } \right\} $$

where the Kalman gain ${\mathbf{K}}(t)$ and observation prediction error ${\mathbf{S}}(t)$ are given by

15 $$ \left. \begin{gathered} {\mathbf{K}}(t) = {{\varvec{\Gamma}}}^{(10)} (t - 1){\mathbf{H}}_{NDT}^{T} {\mathbf{S}}^{ - 1} (t) \hfill \\ {\mathbf{S}}(t) = {\mathbf{H}}_{NDT} {{\varvec{\Gamma}}}^{(10)} (t - 1){\mathbf{H}}_{NDT}^{T} + {\mathbf{R}}_{NDT} \hfill \\ \end{gathered} \right\} $$

where $${\mathbf{R}}_{NDT}^{{}}$$ is the covariance of $$\Delta {\mathbf{z}}_{NDT}$$. In the experiment in Sect. 6, $${\mathbf{R}}_{NDT}^{{}} = {\text{diag}} [(0.01)^{2} ,(0.01)^{2} ,(0.01)^{2} ,(0.01)^{2}$$$$(0.01)^{2} ,$$$$(0.001)^{2} ]$$ is used.
